# Alcohol Use and Sustained Virologic Response to Hepatitis C Virus Direct-Acting Antiviral Therapy

**DOI:** 10.1001/jamanetworkopen.2023.35715

**Published:** 2023-09-26

**Authors:** Emily J. Cartwright, Chloe Pierret, Caroline Minassian, Denise A. Esserman, Janet P. Tate, Matthew B. Goetz, Debika Bhattacharya, David A. Fiellin, Amy C. Justice, Vincent Lo Re, Christopher T. Rentsch

**Affiliations:** 1 Division of Infectious Diseases, Emory University School of Medicine, Atlanta, Georgia; 2 Atlanta Veterans Affairs Medical Center, Decatur, Georgia; 3 Faculty of Epidemiology and Population Health, London School of Hygiene & Tropical Medicine, London, United Kingdom; 4 Veterans Affairs Connecticut Healthcare System, US Department of Veterans Affairs, West Haven; 5 Department of Internal Medicine, Yale School of Medicine, New Haven, Connecticut; 6 Department of Internal Medicine, David Geffen School of Medicine, University of California, Los Angeles; 7 Veterans Affairs Greater Los Angeles Health Care System, US Department of Veterans Affairs, Los Angeles, California; 8 Yale Program in Addiction Medicine, Yale School of Medicine, New Haven, Connecticut; 9 Yale School of Public Health, New Haven, Connecticut; 10 Division of Infectious Diseases, Department of Medicine and Center for Clinical Epidemiology and Biostatistics, Perelman School of Medicine, University of Pennsylvania, Philadelphia

## Abstract

**Importance:**

Some payers and clinicians require alcohol abstinence to receive direct-acting antiviral (DAA) therapy for chronic hepatitis C virus (HCV) infection.

**Objective:**

To evaluate whether alcohol use at DAA treatment initiation is associated with decreased likelihood of sustained virologic response (SVR).

**Design, Setting, and Participants:**

This retrospective cohort study used electronic health records from the US Department of Veterans Affairs (VA), the largest integrated national health care system that provides unrestricted access to HCV treatment. Participants included all patients born between 1945 and 1965 who were dispensed DAA therapy between January 1, 2014, and June 30, 2018. Data analysis was completed in November 2020 with updated sensitivity analyses performed in 2023.

**Exposure:**

Alcohol use categories were generated using responses to the Alcohol Use Disorders Identification Test–Consumption (AUDIT-C) questionnaire and *International Classification of Diseases, Ninth Revision* and *International Statistical Classification of Diseases and Related Health Problems, Tenth Revision *diagnoses for alcohol use disorder (AUD): abstinent without history of AUD, abstinent with history of AUD, lower-risk consumption, moderate-risk consumption, and high-risk consumption or AUD.

**Main Outcomes and Measures:**

The primary outcome was SVR, which was defined as undetectable HCV RNA for 12 weeks or longer after completion of DAA therapy. Multivariable logistic regression was used to estimate odds ratios (ORs) and 95% CIs of SVR associated with alcohol category.

**Results:**

Among 69 229 patients who initiated DAA therapy (mean [SD] age, 62.6 [4.5] years; 67 150 men [97.0%]; 34 655 non-Hispanic White individuals [50.1%]; 28 094 non-Hispanic Black individuals [40.6%]; 58 477 individuals [84.5%] with HCV genotype 1), 65 355 (94.4%) achieved SVR. A total of 32 290 individuals (46.6%) were abstinent without AUD, 9192 (13.3%) were abstinent with AUD, 13 415 (19.4%) had lower-risk consumption, 3117 (4.5%) had moderate-risk consumption, and 11 215 (16.2%) had high-risk consumption or AUD. After adjustment for potential confounding variables, there was no difference in SVR across alcohol use categories, even for patients with high-risk consumption or AUD (OR, 0.95; 95% CI, 0.85-1.07). There was no evidence of interaction by stage of hepatic fibrosis measured by fibrosis-4 score (*P* for interaction = .30).

**Conclusions and Relevance:**

In this cohort study, alcohol use and AUD were not associated with lower odds of SVR. Restricting access to DAA therapy according to alcohol use creates an unnecessary barrier to patients and challenges HCV elimination goals.

## Key Points

**Question:**

Is alcohol use associated with achieving a sustained virologic response (SVR) when treating hepatitis C virus (HCV) infection with direct-acting antiviral (DAA) therapy?

**Findings:**

In this cohort study of 69 229 adults with HCV infection, there was no difference in SVR across alcohol use categories, even for patients with high-risk consumption or alcohol use disorder, after adjusting for potential confounding variables.

**Meaning:**

These findings suggest that restricting access to DAA therapy on the basis of alcohol use creates an unnecessary barrier for patients and challenges HCV elimination goals.

## Teaser

This cohort study evaluates whether alcohol use is associated with achieving a sustained virologic response when treating hepatitis C virus (HCV) infection with direct-acting antiviral therapy.

## Introduction

Previously, chronic hepatitis C virus (HCV) infection was treated with interferon-based regimens, a poorly tolerated therapy. In the interferon era, patients with active alcohol use in the previous year were more likely to discontinue interferon-based HCV treatment; consequently, many clinicians were reluctant to treat those with recent alcohol use.^1^ However, for patients who successfully completed interferon-based therapy, comparable rates of sustained virologic response (SVR) were achieved regardless of reported alcohol use.^1,2,3,4^ In 2009, the American Association for the Study of Liver Diseases (AASLD) treatment guidelines stated that candidates for HCV treatment should be abstinent from alcohol for a minimum of 6 months before initiating treatment.^5^

With the advent of safe and highly effective direct-acting antiviral (DAA) therapy for HCV, the impact of alcohol use on achieving SVR is less clear. Clinical trials^6,7,8,9,10,11^ examining DAA safety and efficacy excluded participants who had clinically relevant alcohol use or reported high-risk alcohol consumption within the prior 12 months, as defined by Alcohol Use Disorder Identification Test–Consumption (AUDIT-C) scores of 8 or more. Empirical data on the effectiveness of DAAs in persons with current unhealthy alcohol use has been limited to an observational study^12^ examining SVR in a cohort of 17 487 US Veterans who initiated DAA therapy between 2014 and mid-2015. The study found that most persons across all alcohol use categories achieved SVR (>91%), and no association between alcohol use and SVR was observed in all primary analyses. However, that study used patients who reported abstinence from alcohol as the referent group, which may be prone to biased results owing to this being a heterogeneous group known to include persons who consumed alcohol previously and quit because of alcohol-related or other health problems (ie, *sick quitters*).^13^

Current AASLD/Infectious Diseases Society of American (IDSA) HCV treatment guidelines advise that patients with HCV avoid excess alcohol use but do not recommend restricting access to DAA therapy on the basis of alcohol intake, regardless of any level of consumption.^14^ Similarly, the Department of Veterans Affairs (VA)—the largest provider of HCV care in the US—does not recommend withholding DAA therapy from patients with alcohol use disorder (AUD). DAA therapy is available to patients with HCV infection at no or substantially reduced cost to the individual in the VA system.^15,16^ Despite these recommendations, some clinicians continue to delay or withhold HCV therapy from patients who consume alcohol.^17,18,19,20^ Furthermore, some payers include alcohol abstinence as a requirement for reimbursement of DAA therapy for HCV.^21,22^ To provide updated, empirical data on the effect of alcohol use on cure of HCV infection, we used national VA electronic health record data to examine the association of alcohol use categories with SVR.

## Methods

### Study Design and Data Source

We conducted a retrospective cohort study using electronic health record data from the VA. The VA comprises more than 1200 points of health care mostly in the US, including hospitals, medical centers, and community outpatient clinics, from which all care is recorded in a central data repository, with daily uploads into the VA Corporate Data Warehouse. The VA Corporate Data Warehouse includes information on demographics, outpatient and inpatient encounters, *International Classification of Diseases, Ninth Revision* and *International Statistical Classification of Diseases and Related Health Problems, Tenth Revision* diagnostic codes, smoking and alcohol use, pharmacy dispensing records, laboratory measures, vital signs, and death. This study was approved by the institutional review boards of Yale University and VA Connecticut Healthcare System. It has been granted a waiver of informed consent because the data were anonymous, in accordance with 45 CFR §46, and is compliant with the Health Insurance Portability and Accountability Act. This study is reported as per the Strengthening the Reporting of Observational Studies in Epidemiology (STROBE) reporting guideline and the Reporting of Studies Conducted Using Observational Routinely-Collected Health Data (RECORD) statements.

### Study Population

We extracted data from the 1945 to 1965 VA Birth Cohort, which includes all individuals born between 1945 and 1965 who had at least 1 VA encounter on or after October 1, 1999. This birth cohort was chosen because people born between 1945 and 1965 are known to have a 6-fold higher prevalence of HCV infection compared with all other age groups.^23^ In addition, the Centers for Disease Control and Prevention and the US Preventive Services Task Force recommended onetime HCV screening for these individuals during the time of study.^24,25^ We included all patients who initiated interferon-free DAA therapy between January 1, 2014, and June 30, 2018. The index date was defined as the day the patient was dispensed their first DAA regimen. We have previously demonstrated that DAA therapies and date of initiation are accurately recorded in VA electronic health record data.^26^

### Alcohol Category

Our primary exposure variable combined information on alcohol consumption (using the AUDIT-C) and AUD diagnoses ascertained in the 18 months before the index date. For those with more than 1 AUDIT-C measure in the ascertainment window, the value closest to the index date was chosen. The AUDIT-C is an externally validated 3-item questionnaire that assesses alcohol consumption and has been required annually in the VA since 2007.^27,28,29,30^ We classified patients as having AUD by the presence of at least 1 inpatient or outpatient diagnostic code, including *International Classification of Diseases, Ninth Revision* and *International Statistical Classification of Diseases and Related Health Problems, Tenth Revision* codes 303.*, 305.0*, F10.1*, or F10.2*.

Consistent with a recent study on DAA initiation in the VA,^20^ we classified patients into 5 mutually exclusive groups on the basis of AUDIT-C score and AUD diagnoses: (1) abstinent without AUD (AUDIT-C score of 0 and absence of AUD diagnosis); (2) abstinent with AUD (AUDIT-C score of 0 and presence of AUD diagnosis); (3) lower-risk consumption (AUDIT-C score of 1-3 and absence of AUD diagnosis); (4) moderate-risk consumption (AUDIT-C score of 4-7 and absence of AUD diagnosis); and (5) high-risk consumption or AUD (AUDIT-C score of ≥8 or presence of AUD diagnosis with nonzero AUDIT-C score). Similar to Haque et al,^20^ we used lower-risk consumption as the referent group in all analyses.

### Outcome

The primary outcome was SVR, defined by undetectable HCV RNA 12 weeks or longer and less than 6 months after completion of DAA therapy. HCV RNA measurements through December 31, 2018, were extracted for analysis. If there were multiple HCV RNA measurements available, the latest result was chosen. For patients with no measurements 12 weeks or more after completion of DAA therapy, we selected the latest result available in the 4 to 11 weeks after completion of DAA therapy. Previous studies^31,32^ have demonstrated high concordance between SVR defined at 4, 12, and 24 weeks after treatment.

### Covariates

**Figure 1.**
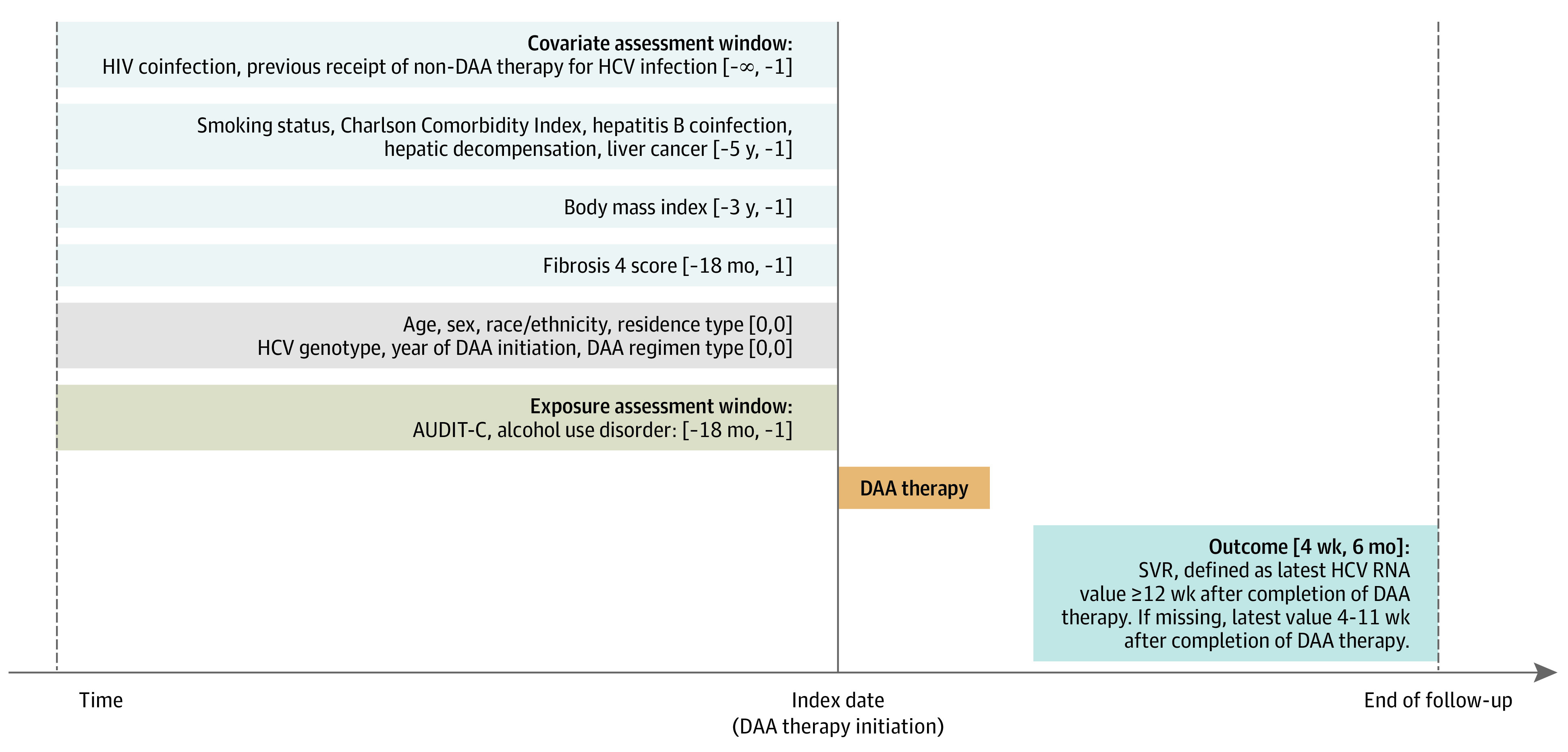
Study Diagram AUDIT-C indicates Alcohol Use Disorder Identification Test–Consumption; DAA, direct-acting antiviral; HCV, hepatitis C virus; and SVR, sustained virologic response.

Figure 1 depicts the study design and details on exposure and covariate ascertainment windows. Covariates included age in years, sex (male vs female), race and ethnicity (Hispanic, non-Hispanic Black, non-Hispanic White, other [ie, American Indian, Asian, multiracial, and Pacific Islander], or missing as identified in the electronic health record), rural or urban residence type, body mass index, smoking status, HIV coinfection, Charlson Comorbidity Index score, liver-related variables (ie, HCV genotype, hepatitis B coinfection, fibrosis 4 [FIB-4] score, hepatic decompensation, or liver cancer), and other HCV treatment-related variables (ie, previous receipt of non-DAA therapy for HCV infection, year of DAA therapy initiation, and DAA regimen type). Data on race and ethnicity were included because of known associations with DAA medication receipt and SVR outcomes.

### Statistical Analysis

 Data analysis was completed in November 2020 with updated sensitivity analyses performed in 2023. We calculated absolute standardized mean differences (SMDs) to identify differences in the covariate distribution between patients with and without AUDIT-C or HCV RNA results to define SVR. We considered SMD less than 0.2 as balanced.^33^

After excluding those with missing alcohol and HCV RNA results, an additional 7339 participants (9.6%) had missing data for any of the other covariates (ie, 6.9% missing FIB-4, 1.4% missing HCV genotype, 1.1% missing smoking status, and 0.8% missing body mass index). Our primary analyses were performed on complete cases because the overall level of missingness among covariates was less than 10%, and a large proportion of missingness was likely to be missing not at random. A complete case analysis will be unbiased if, conditional on model covariates, missingness is independent of the outcome,^34^ which we demonstrate is the case in our data.

We used logistic regression to estimate odds ratios (ORs) and 95% CIs for the association of alcohol category with SVR, adjusting for demographics, clinical characteristics, liver-related variables, and other HCV treatment-related variables. We then assessed possible interaction by specifying a cross-product term in the fully adjusted model between alcohol category and FIB-4 score because FIB-4 scores greater than 3.25 are associated with advanced liver fibrosis or cirrhosis and risk of liver cancer, which may be associated with a lower likelihood of SVR.^35,36^ Statistical significance was set at 2-sided *P* < .05. Analyses were performed using Stata statistical software version 17.0 (StataCorp).

In sensitivity analyses, we compared estimates from the fully adjusted model in the primary analysis to models including patients with missing outcome data using 3 assumptions: (1) multiple imputation (10 imputations) of the outcome with imputation model included all extracted covariates; (2) all patients with missing outcome data were assumed to have achieved SVR; and (3) all patients with missing outcome data were assumed to not have achieved SVR. Although multiple imputation may not be appropriate because it assumes data to define SVR were missing at random,^37^ we included this sensitivity analysis to compare our findings with those of previous studies that applied multiple imputation. We extended the outcome ascertainment window to include HCV RNA measurements up to 12 months after completion of DAA therapy. We reclassified women with AUDIT-C score of 3 and no AUD as having moderate-risk consumption.

## Results

### Cohort Description

**Figure 2.**
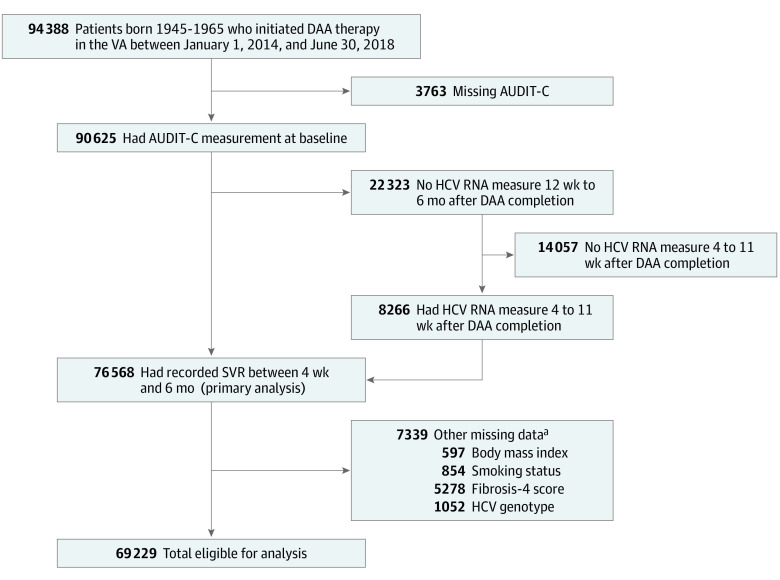
Patient Flow Diagram AUDIT-C indicates Alcohol Use Disorder Identification Test–Consumption; DAA, direct acting antiviral; HCV, hepatitis C virus; SVR, sustained virologic response; and VA, US Department of Veterans Affairs. ^a^Participants could be identified in more than 1 exclusion criteria within each broad exclusion.

Among 94 388 patients who initiated DAA therapy between January 1, 2014, and June 30, 2018 (Figure 2), 3763 (4.0%) had missing AUDIT-C score and 22 323 (24.6%) had no HCV RNA laboratory values to define SVR within 12 weeks to 6 months after DAA completion. Among those with missing HCV RNA laboratory values in this window, 8266 (37.0%) had measurements in the 4 to 11 weeks after completion of DAA therapy; thus, 14 057 patients had no information to define SVR in primary analyses. Compared with patients with AUDIT-C values, Charlson Comorbidity Index scores were lower in patients without AUDIT-C values (SMD = 0.29), but the distribution of other covariates was similar (all SMD <0.2; most SMD <0.1) (eTable 1 in Supplement 1). Distributions were also similar for those with and without HCV RNA laboratory values, including no observed differences by alcohol category (eTable 2 in Supplement 1).

Of the 69 229 patients included in the primary analyses (mean [SD] age, 62.6 [4.5] years), 67 150 (97.0%) were men, 34 655 (50.1%) were non-Hispanic White, 28 094 (40.6%) were non-Hispanic Black, and 46 220 (66.8%) were current smokers at DAA initiation (Table 1). Most patients (58 477 patients [84.5%]) had HCV genotype 1, 15.4% (10 632 patients) had previous receipt of non-DAA therapy for HCV infection, 3.2% (2217 patients) had HIV coinfection, and 1.9% (1308 patients) had HBV coinfection. Approximately one-half of patients (35 032 patients [50.6%]) had FIB-4 scores between 1.45 and 3.25, whereas 16 103 patients (23.3%) had scores less than 1.45, and 18 094 patients (26.1%) had scores greater than 3.25 at DAA initiation. Sofosbuvir and ledipasvir (58.8%) was the most frequently prescribed DAA regimen.

**Table 1.** Characteristics of Patients Who Initiated DAA Therapy by Alcohol Consumption Category

Characteristic	Patients, No. (%) (N = 69 229)				
	Abstinent		Consumption		
	Without AUD (n = 32 290)	With AUD (n = 9192)	Lower risk (n = 13 415)	Moderate risk (n = 3117)	High risk or AUD (n = 11 215)
Demographic characteristics					
Age, median (IQR), y	63.3 (59.9-66.4)	61.6 (58.4-65.0)	63.0 (59.7-66.1)	63.0 (59.8-66.1)	61.8 (58.6-65.2)
48-54	1440 (4.5)	725 (7.9)	662 (4.9)	143 (4.6)	793 (7.1)
55-59	6787 (21.0)	2635 (28.7)	2982 (22.2)	688 (22.1)	3212 (28.6)
60-64	12 418 (38.5)	3497 (38.0)	5233 (39.0)	1248 (40.0)	4290 (38.3)
65-73	11 645 (36.1)	2335 (25.4)	4538 (33.8)	1038 (33.3)	2920 (26.0)
Sex					
Women	1074 (3.3)	251 (2.7)	474 (3.5)	49 (1.6)	231 (2.1)
Men	31 216 (96.7)	8941 (97.3)	12 941 (96.5)	3068 (98.4)	10 984 (97.9)
Race and ethnicity					
Hispanic	1649 (5.1)	450 (4.9)	638 (4.8)	112 (3.6)	506 (4.5)
Non-Hispanic Black	12 702 (39.3)	3738 (40.7)	5608 (41.8)	1117 (35.8)	4929 (44.0)
Non-Hispanic White	16 403 (50.8)	4666 (50.8)	6535 (48.7)	1723 (55.3)	5.327 (47.5)
Other or missing^a^	1536 (4.8)	338 (3.7)	633 (4.7)	165 (5.3)	453 (4.0)
Residence type					
Rural	8815 (27.3)	2099 (22.8)	3516 (26.2)	964 (30.9)	2695 (24.0)
Urban	23 475 (72.7)	7093 (77.2)	9899 (73.8)	2153 (69.1)	8520 (76.0)
Clinical characteristics					
Body mass index^b^					
Underweight (<18.5)	467 (1.4)	126 (1.4)	208 (1.6)	58 (1.9)	243 (2.2)
Normal (18.5-24.9)	8482 (26.3)	2601 (28.3)	3783 (28.2)	1084 (34.8)	3952 (35.2)
Overweight (25.0-29.9)	12 244 (37.9)	3515 (38.2)	5232 (39.0)	1186 (38.0)	4208 (37.5)
Obese (≥30.0)	11 097 (34.4)	2950 (32.1)	4192 (31.2)	789 (25.3)	2812 (25.1)
Smoking status					
Never	5117 (15.8)	795 (8.6)	1840 (13.7)	323 (10.4)	779 (6.9)
Current	18 686 (57.9)	7053 (76.7)	8831 (65.8)	2305 (73.9)	9345 (83.3)
Former	8487 (26.3)	1344 (14.6)	2744 (20.5)	489 (15.7)	1091 (9.7)
HIV coinfection	1072 (3.3)	318 (3.5)	409 (3.0)	74 (2.4)	344 (3.1)
Charlson Comorbidity Index score					
1	12 107 (37.5)	3322 (36.1)	6031 (45.0)	1578 (50.6)	4883 (43.5)
2	8230 (25.5)	2394 (26.0)	3398 (25.3)	834 (26.8)	2976 (26.5)
3	3273 (10.1)	879 (9.6)	1284 (9.6)	265 (8.5)	1072 (9.6)
4	3313 (10.3)	913 (9.9)	1098 (8.2)	195 (6.3)	921 (8.2)
≥5	5367 (16.6)	1684 (18.3)	1604 (12.0)	245 (7.9)	1363 (12.2)
Liver-related variables					
HCV genotype					
1	27 342 (84.7)	7727 (84.1)	11 358 (84.7)	2584 (82.9)	9466 (84.4)
2	2889 (8.9)	824 (9.0)	1242 (9.3)	354 (11.4)	1023 (9.1)
3	1739 (5.4)	560 (6.1)	663 (4.9)	153 (4.9)	610 (5.4)
4, 5, or 6	320 (1.0)	81 (0.9)	152 (1.1)	26 (0.8)	116 (1.0)
Hepatitis B coinfection	581 (1.8)	253 (2.8)	185 (1.4)	43 (1.4)	246 (2.2)
Fibrosis-4 score					
<1.45	7402 (22.9)	2162 (23.5)	3400 (25.3)	712 (22.8)	2427 (21.6)
1.45-3.25	16 489 (51.1)	4353 (47.4)	7137 (53.2)	1641 (52.6)	5412 (48.3)
>3.25	8399 (26.0)	2677 (29.1)	2878 (21.5)	764 (24.5)	3376 (30.1)
Hepatic decompensation	1091 (3.4)	640 (7.0)	117 (0.9)	14 (0.4)	346 (3.1)
Liver cancer	786 (2.4)	363 (3.9)	154 (1.1)	34 (1.1)	200 (1.8)
Treatment-related characteristics					
Previous non-DAA therapy for HCV infection	6021 (18.6)	1551 (16.9)	1635 (12.2)	298 (9.6)	1127 (10.0)
Year of DAA therapy initiation					
2014	3298 (10.2)	978 (10.6)	760 (5.7)	116 (3.7)	466 (4.2)
2015	9995 (31.0)	3111 (33.8)	3556 (26.5)	584 (18.7)	2623 (23.4)
2016	11 574 (35.8)	3325 (36.2)	5204 (38.8)	1271 (40.8)	4502 (40.1)
2017	5932 (18.4)	1400 (15.2)	3099 (23.1)	923 (29.6)	2826 (25.2)
2018	1491 (4.6)	378 (4.1)	796 (5.9)	223 (7.2)	798 (7.1)
DAA regimen type					
Sofosbuvir and ledipasvir	18 526 (57.4)	5569 (60.6)	7954 (59.3)	1867 (59.9)	6805 (60.7)
Elbasvir and grazoprevir	3387 (10.5)	729 (7.9)	1484 (11.1)	361 (11.6)	1234 (11.0)
Sofosbuvir and velpatasvir with or without voxilaprevir	2270 (7.0)	592 (6.4)	1085 (8.1)	341 (10.9)	1000 (8.9)
Glecaprevir and pibrentasvir	972 (3.0)	234 (2.5)	552 (4.1)	127 (4.1)	506 (4.5)
Paritaprevir, ritonavir, and ombitasvir with or without dasabuvir	2747 (8.5)	697 (7.6)	1084 (8.1)	199 (6.4)	725 (6.5)
Sofosbuvir and daclatasvir	389 (1.2)	121 (1.3)	148 (1.1)	28 (0.9)	124 (1.1)
Simeprevir and sofosbuvir	1410 (4.4)	427 (4.6)	295 (2.2)	29 (0.9)	205 (1.8)
Sofosbuvir and ribavirin	2589 (8.0)	823 (9.0)	813 (6.1)	165 (5.3)	616 (5.5)

Abbreviations: AUD, alcohol use disorder; DAA, direct-acting antiviral; HCV, hepatitis C virus.

^a^ Other includes American Indian, Asian, multiracial, and Pacific Islander.

^b^ Body mass index is calculated as weight in kilograms divided by height in meters squared.

### Alcohol Category and SVR

There was considerable variation in the alcohol category: 32 290 patients (46.6%) were abstinent without AUD, 9192 patients (13.3%) were abstinent with AUD, 13 415 patients (19.4%) had lower-risk consumption, 3117 patients (4.5%) had moderate-risk consumption, and 11 215 patients (16.2%) had high-risk consumption or AUD. Overall, 65 355 (94.4%) of all DAA-initiating patients achieved SVR, with 58 651 SVR outcomes measured 12 weeks to 6 months after DAA completion, and 6704 at 4 to 12 weeks after DAA completion.

In an unadjusted model, patients who were abstinent without AUD history (OR, 0.89 [95% CI, 0.81-0.97]) and those who were abstinent with AUD history (OR, 0.71 [95% CI, 0.64-0.80]) had lower odds of achieving SVR compared with patients who reported lower-risk consumption (Table 2). However, in the fully adjusted model, we found no evidence that any alcohol category was significantly associated with decreased odds of SVR (OR, 1.09 [95% CI, 0.99-1.20] for abstinent without AUD history; OR, 0.92 [95% CI, 0.82-1.04] for abstinent with AUD history; OR, 0.96 [95% CI, 0.80-1.15] for moderate-risk consumption; and OR, 0.95 [95% CI, 0.85-1.07] for high-risk consumption or AUD) (Table 2). In addition, we found no evidence that the association of alcohol category with the odds of SVR differed by baseline stage of hepatic fibrosis measured by FIB-4 less than or equal to 3.25 vs greater than 3.25 (*P* for interaction = .30) (Table 2).

**Table 2.** Associations of Alcohol Category With Sustained Virologic Response

Variable	OR (95% CI)^a^				
	All patients with exposure and outcome data (n = 76 568), unadjusted	Complete case analysis (n = 69 229)		By fibrosis-4 score^b^	
		Unadjusted	Fully adjusted	≤3.25 (n = 51 135)	>3.25 (n = 18 094)
Abstinent					
Without AUD	0.88 (0.80-0.95)	0.89 (0.81-0.97)	1.09 (0.99-1.20)	1.11 (0.99-1.25)	1.08 (0.92-1.27)
With AUD	0.70 (0.63-0.78)	0.71 (0.64-0.80)	0.92 (0.82-1.04)	0.96 (0.82-1.11)	0.88 (0.72-1.06)
Consumption					
Lower risk	1 [Reference]	1 [Reference]	1 [Reference]	1 [Reference]	1 [Reference]
Moderate risk	1.03 (0.87-1.22)	1.04 (0.86-1.24)	0.96 (0.80-1.15)	1.00 (0.79-1.26)	0.87 (0.64-1.19)
High risk or AUD	0.91 (0.81-1.01)	0.92 (0.82-1.03)	0.95 (0.85-1.07)	0.91 (0.79-1.06)	1.02 (0.82-1.21)

Abbreviations: AUD, alcohol use disorder; OR, odds ratio.

^a^ Fully adjusted model included age, sex, race and ethnicity, residence type, body mass index, smoking status, HIV coinfection, Charlson Comorbidity Index score, liver-related variables (ie, hepatitis C virus [HCV] genotype, hepatitis B coinfection, fibrosis-4 score, hepatic decompensation, and liver cancer), and other HCV treatment-related variables (ie, previous receipt of non–direct-acting antiviral [DAA] therapy for HCV infection, year of DAA therapy initiation, and DAA regimen type).

^b^ *P* value for interaction = .30.

### Sensitivity Analyses

Findings persisted after including patients with missing data using multiple imputation (lowest adjusted OR, 0.90 [95% CI, 0.80-1.02] for patients who were abstinent with AUD history) and assuming all patients with missing outcome data achieved SVR (lowest adjusted OR, 0.94 [95% CI, 0.84-1.06] for patients who were abstinent with AUD history) (Table 3). In an analysis assuming the highly unlikely scenario that all patients with missing outcome data did not achieve SVR, we observed that patients who were abstinent with AUD history (adjusted OR, 0.92 [95% CI, 0.86-0.98]) and those with high-risk consumption or AUD (adjusted OR, 0.88 [95% CI, 0.83-0.93]) had lower odds of SVR compared with patients who reported lower-risk consumption. Conclusions from the primary analysis held after extending the outcome ascertainment window from 6 to 12 months. Results were nearly identical after reclassifying women with AUDIT-C score of 3 and no AUD as moderate risk consumption.

**Table 3.** Sensitivity Analyses

Variable	Adjusted OR (95% CI)^a^				
	Primary analysis (n = 69 229)	Multiple imputation of missing SVR (n = 81 703)	Missing SVR, assumed to be cured (n = 81 703)	Missing SVR, assumed to be not cured (n = 81 703)	Extending outcome ascertainment window (n = 73 956)
Abstinent					
Without AUD	1.09 (0.99-1.20)	1.08 (0.97-1.20)	1.09 (0.99-1.20)	1.00 (0.96-1.05)	1.08 (0.96-1.20)
With AUD	0.92 (0.82-1.04)	0.90 (0.80-1.02)	0.94 (0.84-1.06)	0.92 (0.86-0.98)	0.95 (0.83-1.09)
Consumption					
Lower risk	1 [Reference]	1 [Reference]	1 [Reference]	1 [Reference]	1 [Reference]
Moderate risk	0.96 (0.80-1.15)	0.95 (0.76-1.19)	0.95 (0.79-1.15)	1.03 (0.94-1.13)	0.98 (0.78-1.22)
High risk or AUD	0.95 (0.85-1.07)	0.95 (0.84-1.08)	0.98 (0.87-1.11)	0.88 (0.83-0.93)	0.96 (0.84-1.10)

Abbreviations: AUD, alcohol use disorder; OR, odds ratio; SVR, sustained virologic response.

^a^ Fully adjusted model included age, sex, race and ethnicity, residence type, body mass index, smoking status, HIV coinfection, Charlson Comorbidity Index score, liver-related variables (ie, hepatitis C virus [HCV] genotype, hepatitis B coinfection, fibrosis-4 score, hepatic decompensation, and liver cancer), and other HCV treatment-related variables (ie, previous receipt of non–direct-acting antiviral [DAA] therapy for HCV infection, year of DAA therapy initiation, and DAA regimen type).

## Discussion

In this nationwide cohort study of chronically HCV-infected adults born between 1945 and 1965 who initiated DAA therapy between 2014 and 2018 in the largest provider of HCV care in the US, we found no evidence that any level of alcohol use was associated with decreased odds of achieving SVR. This finding did not differ by baseline stage of hepatic fibrosis. Moreover, 94.4% of all patients achieved SVR in this clinical practice setting of an older population with multiple comorbidities who are typically underrepresented in clinical trials. Furthermore, some patients were treated with older DAA regimens including sofosbuvir and ribavirin, which are less well tolerated and are associated with a lower percentage of patients achieving SVR. Taken together, our findings support providing DAA therapy without regard to reported alcohol consumption or AUD.

Our results support the current AASLD/IDSA recommendations that current or prior alcohol use is not a contraindication to HCV DAA therapy. However, a recent analysis of administrative claims and encounters in the US found that only 23% of Medicaid, 28% of Medicare, and 35% of private insurance recipients initiated DAA treatment within 1 year of HCV diagnosis.^38^ Furthermore, that analysis found that Medicaid recipients were less likely to initiate DAA treatment if they resided in a state with Medicaid treatment restrictions including alcohol abstinence. Even though the VA has not required alcohol abstinence before DAA treatment, preexisting alcohol use still impacts who initiates DAA therapy. Haque et al^20^ found that individuals in the 1945 to 1965 VA Birth Cohort with current AUD and those who were abstinent with an AUD history were significantly less likely to receive DAA treatment compared with patients with lower-risk drinking. Although alcohol use has a dose-dependent association with incident cirrhosis and is associated with increased risk of hepatocellular carcinoma in those with chronic HCV infection, curative HCV treatment has been shown to significantly reduce the risk of cirrhosis and hepatocellular carcinoma in those with chronic hepatitis C.^39,40,41^ The VA does offer substance use disorder treatment, and any veteran can be referred and assessed for treatment, which may include inpatient, intensive outpatient, university model, or residential treatment programs. Some VA facilities partnered with residential substance use disorder program to integrate HCV treatment.^42^ Our findings suggest that clinicians and policy makers should encourage HCV treatment in those with unhealthy alcohol consumption or AUD, rather than creating barriers to HCV treatment. Given the high rates of SVR across all alcohol use categories, there is no indication for payers to require alcohol abstinence before reimbursement of DAA therapy for HCV infection.

Although numerous studies from the interferon era examined alcohol use and SVR,^1,2,3,4^ there are few such studies in the DAA era. Tsui et al^12^ examined alcohol use and SVR in the VA including the first 18 months of the DAA era. Although the authors found no difference in SVR across alcohol use categories in all primary analyses, after imputing missing HCV RNA values for 9% of their cohort, they observed a significantly lower likelihood of SVR among those reporting unhealthy alcohol use. However, Tsui et al^12^ also demonstrated that those with unhealthy alcohol use (defined as AUDIT-C score ≥4) were more likely to have missing HCV RNA values, and, therefore, the assumptions required for multiple imputation may have been violated.^37^ Furthermore, Tsui et al^12^ used patients reporting abstinence as the referent group; however, this is a heterogeneous group comprising very few lifetime abstainers, and the majority quit drinking after experiencing alcohol-related or health problems.^13^ To avoid misclassification, we used lower-risk consumption as the referent group and distinguished patients reporting abstinence by whether they had received a diagnosis of AUD. In sensitivity analyses assuming everyone with missing outcome data did not achieve SVR, persons with high-risk consumption or AUD had 12% decreased odds of achieving SVR (adjusted OR, 0.88) in multivariable analysis. We know in clinical practice that not every missing SVR value is a treatment failure, and, in fact, many persons are found to have SVR when reengaged in care^43^; therefore, we express caution in interpreting this single association.

### Strengths and Limitations

This study has many strengths, including the availability of detailed, longitudinal, electronic health record data on a diverse nationwide cohort of HCV-infected patients initiating DAA therapy and findings that were robust to multiple sensitivity analyses. Importantly, the VA has been more successful than most health care systems in the US in diagnosing and treating HCV infection given its unified electronic health record, nationalized health care system, and prioritization of HCV.^44^ The VA has far exceeded other health care systems^38,45,46,47,48,49^ by treating nearly 85% of patients with known chronic HCV infection in VA care.^16,50,51^

We also recognize possible limitations. First, as a result of the observational nature of the study, a degree of uncertainty persists owing to the potential for residual confounding. Second, we assessed HCV RNA measurements in the 6-month period following the end of DAA treatment to define SVR, which may have resulted in potential misclassification of some patients who may have experienced viral relapse; however, this has been shown to be an extremely rare event.^52^ Some patients may also have had their first evidence of SVR recorded beyond that window of measurement, which we would have classified as a missing outcome, although extending the ascertainment window to 12 months did not change our results. Third, alcohol use measurement may have been influenced by both patient-level and practitioner-level factors,^53,54,55,56^ including underreporting level of alcohol use because of social desirability bias,^57^ which may have resulted in misclassification of some patients with high-risk consumption at lower levels of consumption. Furthermore, we categorized patients with AUD separately and used patients with lower-risk consumption as the referent category, which minimized the potential of misclassification of sick quitters.^13^ Fourth, we performed a complete case analysis, which will be unbiased if, conditional on model covariates, missingness is independent of the outcome.^34^ To that end, we demonstrated that missing outcome data were not associated with any covariate used in the model, suggesting complete case analysis was appropriate. However, we also performed multiple sensitivity analyses, all of which were consistent with our findings from our complete case analysis. Fifth, our study leveraged available data on patents born between 1945 and 1965; therefore, our findings may not generalize to patients born before 1945 or after 1965. Sixth, although individuals in VA care represent a diversity of backgrounds, women represented a small proportion of individuals in the cohort. However, given the current payer restrictions requiring alcohol abstinence before initiating DAA therapy, the ability to study the association of alcohol use with HCV treatment outcomes, including SVR, may be limited in other US health care settings.

## Conclusions

In conclusion, achieving SVR has been shown to be associated with reduced risk of post-SVR outcomes, including hepatocellular carcinoma, liver-related mortality, and all-cause mortality. Our findings suggest that DAA therapy should be provided and reimbursed despite alcohol consumption or history of AUD. Restricting access to DAA therapy according to alcohol consumption or AUD creates an unnecessary barrier to patients accessing DAA therapy and challenges HCV elimination goals.

## Notes

Supplement 1. **eTable 1.** Covariate Distribution Between Those With and Without Primary Exposure Data **eTable 2.** Covariate Distribution Between Those With and Without Outcome Data

Supplement 2.
Data Sharing Statement

## References

[1] Anand BS, Currie S, Dieperink E, ; VA-HCV-001 Study Group. Alcohol use and treatment of hepatitis C virus: results of a national multicenter study. Gastroenterology. 2006;130(6):1607-1616. doi:10.1053/j.gastro.2006.02.023 16697724

[2] Bruggmann P, Dampz M, Gerlach T, Kravecz L, Falcato L. Treatment outcome in relation to alcohol consumption during hepatitis C therapy: an analysis of the Swiss Hepatitis C Cohort Study. Drug Alcohol Depend. 2010;110(1-2):167-171. doi:10.1016/j.drugalcdep.2010.02.016 20334985

[3] Russell M, Pauly MP, Moore CD, . The impact of lifetime alcohol use on hepatitis C treatment outcomes in privately insured members of an integrated health care plan. Hepatology. 2012;56(4):1223-1230. doi:10.1002/hep.25755 22488513 PMC3426625

[4] Evon DM, Simpson K, Kixmiller S, . A randomized controlled trial of an integrated care intervention to increase eligibility for chronic hepatitis C treatment. Am J Gastroenterol. 2011;106(10):1777-1786. doi:10.1038/ajg.2011.219 21769136 PMC3683982

[5] Ghany MG, Strader DB, Thomas DL, Seeff LB; American Association for the Study of Liver Diseases. Diagnosis, management, and treatment of hepatitis C: an update. Hepatology. 2009;49(4):1335-1374. doi:10.1002/hep.22759 19330875 PMC7477893

[6] Forns X, Lee SS, Valdes J, . Glecaprevir plus pibrentasvir for chronic hepatitis C virus genotype 1, 2, 4, 5, or 6 infection in adults with compensated cirrhosis (EXPEDITION-1): a single-arm, open-label, multicentre phase 3 trial. Lancet Infect Dis. 2017;17(10):1062-1068. doi:10.1016/S1473-3099(17)30496-6 28818546

[7] Zeuzem S, Foster GR, Wang S, . Glecaprevir-pibrentasvir for 8 or 12 weeks in HCV genotype 1 or 3 infection. N Engl J Med. 2018;378(4):354-369. doi:10.1056/NEJMoa1702417 29365309

[8] Feld JJ, Jacobson IM, Hézode C, ; ASTRAL-1 Investigators. Sofosbuvir and velpatasvir for HCV genotype 1, 2, 4, 5, and 6 infection. N Engl J Med. 2015;373(27):2599-2607. doi:10.1056/NEJMoa1512610 26571066

[9] Afdhal N, Zeuzem S, Kwo P, ; ION-1 Investigators. Ledipasvir and sofosbuvir for untreated HCV genotype 1 infection. N Engl J Med. 2014;370(20):1889-1898. doi:10.1056/NEJMoa1402454 24725239

[10] Kowdley KV, Gordon SC, Reddy KR, ; ION-3 Investigators. Ledipasvir and sofosbuvir for 8 or 12 weeks for chronic HCV without cirrhosis. N Engl J Med. 2014;370(20):1879-1888. doi:10.1056/NEJMoa1402355 24720702

[11] Bourlière M, Gordon SC, Flamm SL, ; POLARIS-1 and POLARIS-4 Investigators. Sofosbuvir, velpatasvir, and voxilaprevir for previously treated HCV infection. N Engl J Med. 2017;376(22):2134-2146. doi:10.1056/NEJMoa1613512 28564569

[12] Tsui JI, Williams EC, Green PK, Berry K, Su F, Ioannou GN. Alcohol use and hepatitis C virus treatment outcomes among patients receiving direct antiviral agents. Drug Alcohol Depend. 2016;169:101-109. doi:10.1016/j.drugalcdep.2016.10.021 27810652 PMC6534140

[13] Gordon KS, McGinnis K, Dao C, . Differentiating types of self-reported alcohol abstinence. AIDS Behav. 2020;24(2):655-665. doi:10.1007/s10461-019-02638-x 31435887 PMC6994373

[14] American Association for the Study of Liver Diseases/Infectious Diseases Society of American (AASLD/IDSA). HCV guidance: recommendations for testing, managing, and treating hepatitis C. Accessed March 24, 2023. https://www.hcvguidelines.org/

[15] Belperio PS, Chartier M, Gonzalez RI, . Hepatitis C care in the Department of Veterans Affairs: building a foundation for success. Infect Dis Clin North Am. 2018;32(2):281-292. doi:10.1016/j.idc.2018.02.011 29778256

[16] Yee HS, Burton MJ, Belperio PS, Morgan TR. The Veterans Affairs hepatitis C treatment considerations. Am J Gastroenterol. 2019;114(2):185-188. doi:10.1038/s41395-018-0231-4 30315291

[17] Harris AM, Khan MA, Osinubi A, Nelson NP, Thompson WW. Hepatitis C treatment among commercially or Medicaid-insured individuals, 2014-2018. Am J Prev Med. 2021;61(5):716-723. doi:10.1016/j.amepre.2021.05.017 34362617

[18] Marcus JL, Hurley LB, Chamberland S, . Disparities in initiation of direct-acting antiviral agents for hepatitis C virus infection in an insured population. Public Health Rep. 2018;133(4):452-460. doi:10.1177/0033354918772059 29750893 PMC6055302

[19] Jiang X, Song HJ, Wang W, . The use of all-oral direct-acting antivirals in hepatitis C virus-infected patients with substance use disorders. J Manag Care Spec Pharm. 2021;27(7):873-881. doi:10.18553/jmcp.2021.27.7.873 34185563 PMC8244773

[20] Haque LY, Fiellin DA, Tate JP, . Association between alcohol use disorder and receipt of direct-acting antiviral hepatitis C virus treatment. JAMA Netw Open. 2022;5(12):e2246604. doi:10.1001/jamanetworkopen.2022.46604 36515952 PMC9856353

[21] Martin MT, Waring N, Forrest J, . Sustained virologic response rates before and after removal of sobriety restriction for hepatitis C virus treatment access. Public Health Rep. 2023;138(3):467-474. doi:10.1177/00333549221099323 35674245 PMC10240896

[22] Center for Health Law and Policy Innovation and National Viral Hepatitis Roundtable. 2023 National snapshot report: hepatitis C—state of Medicaid access. Accessed March 24, 2023. https://stateofhepc.org/2023-national-snapshot-report/

[23] Denniston MM, Jiles RB, Drobeniuc J, . Chronic hepatitis C virus infection in the United States, National Health and Nutrition Examination Survey 2003 to 2010. Ann Intern Med. 2014;160(5):293-300. doi:10.7326/M13-1133 24737271 PMC4562398

[24] Smith BD, Morgan RL, Beckett GA, ; Centers for Disease Control and Prevention. Recommendations for the identification of chronic hepatitis C virus infection among persons born during 1945-1965. MMWR Recomm Rep. 2012;61(RR-4):1-32. 22895429

[25] Chou R, Cottrell EB, Wasson N, Rahman B, Guise JM. Screening for hepatitis C virus infection in adults: a systematic review for the U.S. Preventive Services Task Force. Ann Intern Med. 2013;158(2):101-108. doi:10.7326/0003-4819-158-2-201301150-00574 23183613

[26] Rentsch CT, Cartwright EJ, Gandhi NR, . Provider verification of electronic health record receipt and nonreceipt of direct-acting antivirals for the treatment of hepatitis C virus infection. Ann Epidemiol. 2018;28(11):808-811. doi:10.1016/j.annepidem.2018.08.007 30195616 PMC6318448

[27] Bush K, Kivlahan DR, McDonell MB, Fihn SD, Bradley KA. The AUDIT alcohol consumption questions (AUDIT-C): an effective brief screening test for problem drinking. Ambulatory Care Quality Improvement Project (ACQUIP). Alcohol Use Disorders Identification Test. Arch Intern Med. 1998;158(16):1789-1795. doi:10.1001/archinte.158.16.1789 9738608

[28] Fiellin DA, Reid MC, O’Connor PG. Screening for alcohol problems in primary care: a systematic review. Arch Intern Med. 2000;160(13):1977-1989. doi:10.1001/archinte.160.13.1977 10888972

[29] Bradley KA, Williams EC, Achtmeyer CE, Volpp B, Collins BJ, Kivlahan DR. Implementation of evidence-based alcohol screening in the Veterans Health Administration. Am J Manag Care. 2006;12(10):597-606. 17026414

[30] Justice AC, McGinnis KA, Tate JP, . Risk of mortality and physiologic injury evident with lower alcohol exposure among HIV infected compared with uninfected men. Drug Alcohol Depend. 2016;161:95-103. doi:10.1016/j.drugalcdep.2016.01.017 26861883 PMC4792710

[31] Yoshida EM, Sulkowski MS, Gane EJ, . Concordance of sustained virological response 4, 12, and 24 weeks post-treatment with sofosbuvir-containing regimens for hepatitis C virus. Hepatology. 2015;61(1):41-45. doi:10.1002/hep.27366 25314116

[32] Burgess SV, Hussaini T, Yoshida EM. Concordance of sustained virologic response at weeks 4, 12 and 24 post-treatment of hepatitis c in the era of new oral direct-acting antivirals: a concise review. Ann Hepatol. 2016;15(2):154-159. 26845592 10.5604/16652681.1193693

[33] Austin PC. Balance diagnostics for comparing the distribution of baseline covariates between treatment groups in propensity-score matched samples. Stat Med. 2009;28(25):3083-3107. doi:10.1002/sim.3697 19757444 PMC3472075

[34] White IR, Carlin JB. Bias and efficiency of multiple imputation compared with complete-case analysis for missing covariate values. Stat Med. 2010;29(28):2920-2931. doi:10.1002/sim.3944 20842622

[35] Vallet-Pichard A, Mallet V, Nalpas B, . FIB-4: an inexpensive and accurate marker of fibrosis in HCV infection—comparison with liver biopsy and fibrotest. Hepatology. 2007;46(1):32-36. doi:10.1002/hep.21669 17567829

[36] Sakurai T, Kudo M. Molecular link between liver fibrosis and hepatocellular carcinoma. Liver Cancer. 2013;2(3-4):365-366. doi:10.1159/000343851 24400223 PMC3881314

[37] Bhaskaran K, Smeeth L. What is the difference between missing completely at random and missing at random? Int J Epidemiol. 2014;43(4):1336-1339. doi:10.1093/ije/dyu080 24706730 PMC4121561

[38] Thompson WW, Symum H, Sandul A, . Vital signs: hepatitis C treatment among insured adults—United States, 2019-2020. MMWR Morb Mortal Wkly Rep. 2022;71(32):1011-1017. doi:10.15585/mmwr.mm7132e1 35951484 PMC9400534

[39] Llamosas-Falcón L, Shield KD, Gelovany M, . Impact of alcohol on the progression of HCV-related liver disease: a systematic review and meta-analysis. J Hepatol. 2021;75(3):536-546. doi:10.1016/j.jhep.2021.04.018 33892007

[40] Llamosas-Falcón L, Shield KD, Gelovany M, Manthey J, Rehm J. Alcohol use disorders and the risk of progression of liver disease in people with hepatitis C virus infection: a systematic review. Subst Abuse Treat Prev Policy. 2020;15(1):45. doi:10.1186/s13011-020-00287-1 32605584 PMC7325038

[41] Taylor AL, Denniston MM, Klevens RM, McKnight-Eily LR, Jiles RB. Association of hepatitis C virus with alcohol use among U.S. adults: NHANES 2003-2010. Am J Prev Med. 2016;51(2):206-215. doi:10.1016/j.amepre.2016.02.033 27178884

[42] Burton MJ, Voluse AC, Anthony V. Integrating comprehensive hepatitis C virus care within a residential substance use disorder treatment program. J Subst Abuse Treat. 2019;98:9-14. doi:10.1016/j.jsat.2018.11.008 30665610

[43] Backus LI, Belperio PS, Shahoumian TA, Loomis TP, Mole LA. Real-world effectiveness and predictors of sustained virological response with all-oral therapy in 21,242 hepatitis C genotype-1 patients. Antivir Ther. 2017;22(6):481-493. doi:10.3851/IMP3117 27934775

[44] Yakovchenko V, Morgan TR, Chinman MJ, . Mapping the road to elimination: a 5-year evaluation of implementation strategies associated with hepatitis C treatment in the Veterans Health Administration. BMC Health Serv Res. 2021;21(1):1348. doi:10.1186/s12913-021-07312-4 34922538 PMC8684191

[45] Ferrante ND, Newcomb CW, Forde KA, . The hepatitis C care cascade during the direct-acting antiviral era in a United States commercially insured population. Open Forum Infect Dis. 2022;9(9):ofac445. doi:10.1093/ofid/ofac445 36092829 PMC9454032

[46] Ward JW, Hinman AR. What is needed to eliminate hepatitis B virus and hepatitis C virus as global health threats. Gastroenterology. 2019;156(2):297-310. doi:10.1053/j.gastro.2018.10.048 30391470

[47] Maticic M, Pirnat Z, Leicht A, . The civil society monitoring of hepatitis C response related to the WHO 2030 elimination goals in 35 European countries. Harm Reduct J. 2020;17(1):89. doi:10.1186/s12954-020-00439-3 33213481 PMC7678126

[48] Klein MB. Hepatitis C virus elimination: time for disruptive innovation. J Int AIDS Soc. 2019;22(7):e25360. doi:10.1002/jia2.25360 31347280 PMC6658838

[49] Yousafzai MT, Bajis S, Alavi M, Grebely J, Dore GJ, Hajarizadeh B. Global cascade of care for chronic hepatitis C virus infection: a systematic review and meta-analysis. J Viral Hepat. 2021;28(10):1340-1354. doi:10.1111/jvh.13574 34310812

[50] Belperio PS, Chartier M, Ross DB, Alaigh P, Shulkin D. Curing hepatitis C virus infection: best practices from the U.S. Department of Veterans Affairs. Ann Intern Med. 2017;167(7):499-504. doi:10.7326/M17-1073 28973196

[51] Gonzalez R, Park A, Belperio P, . Diagnosis and treatment of HCV in the VA healthcare system. J Acquir Immune Defic Syndr. 2019;81:51. doi:10.1097/01.qai.0000557994.33817.d2

[52] Frías M, Rivero-Juárez A, Téllez F, . Evaluation of hepatitis C viral RNA persistence in HIV-infected patients with long-term sustained virological response by droplet digital PCR. Sci Rep. 2019;9(1):12507. doi:10.1038/s41598-019-48966-9 31467339 PMC6715682

[53] Bradley KA, Lapham GT, Hawkins EJ, . Quality concerns with routine alcohol screening in VA clinical settings. J Gen Intern Med. 2011;26(3):299-306. doi:10.1007/s11606-010-1509-4 20859699 PMC3043188

[54] Lapham GT, Rubinsky AD, Heagerty PJ, . Annual rescreening for alcohol misuse: diminishing returns for some patient subgroups. Med Care. 2013;51(10):914-921. doi:10.1097/MLR.0b013e3182a3e549 23969582

[55] Williams EC, Achtmeyer CE, Thomas RM, . Factors underlying quality problems with alcohol screening prompted by a clinical reminder in primary care: a multi-site qualitative study. J Gen Intern Med. 2015;30(8):1125-1132. doi:10.1007/s11606-015-3248-z 25731916 PMC4510245

[56] McGinnis KA, Tate JP, Williams EC, . Comparison of AUDIT-C collected via electronic medical record and self-administered research survey in HIV infected and uninfected patients. Drug Alcohol Depend. 2016;168:196-202. doi:10.1016/j.drugalcdep.2016.09.015 27694059 PMC5086273

[57] Davis CG, Thake J, Vilhena N. Social desirability biases in self-reported alcohol consumption and harms. Addict Behav. 2010;35(4):302-311. doi:10.1016/j.addbeh.2009.11.001 19932936

