# Supplementary material for: Alcohol Use and Sustained Virologic Response to Hepatitis C Virus Direct-Acting Antiviral Therapy
Source: JAMA Netw Open. 2023 Sep 26;6(9):e2335715. doi: 10.1001/jamanetworkopen.2023.35715 (PMC10523171; doi:10.1001/jamanetworkopen.2023.35715)
Supplement: Supplement 1. — eTable 1. Covariate Distribution Between Those With and Without Primary Exposure Data eTable 2. Covariate Distribution Between Those With and Without Outcome Data [file jamanetwopen-e2335715-s001.pdf]

Supplemental Online Content

Cartwright EJ, Pierret C, Minassian C, et al. Alcohol use and sustained virologic response to hepatitis C virus direct-acting antiviral therapy. *JAMA Netw Open*. 2023;6(9):e2335715. doi:10.1001/jamanetworkopen.2023.35715

eTable 1. Covariate Distribution Between Those With and Without Primary Exposure Data

eTable 2. Covariate Distribution Between Those With and Without Outcome Data

This supplemental material has been provided by the authors to give readers additional information about their work.

© 2023 Cartwright EJ et al. *JAMA Network Open.*

eTable 1. Covariate Distribution Between Those With and Without Primary Exposure Data

	Not missing AUDIT-C n=90,625	Missing AUDIT-C n=3,763	SMD
**Outcome**			
Sustained virologic response			
No	4,279 (4.7)	181 (4.8)	0.07
Yes	72,289 (79.8)	2,900 (77.1)	
Missing	14,057 (15.5)	682 (18.1)	
**Demographics**			
Age in years, median (IQR)			
48-54	5,135 (5.7)	217 (5.8)	0.01
55-59	21,826 (24.1)	903 (24.0)	
60-64	34,841 (38.4)	1,437 (38.2)	
65-73	28,823 (31.8)	1,206 (32.0)	
Sex			
Women	2,714 (3.0)	102 (2.7)	0.02
Men	87,911 (97.0)	3,661 (97.3)	
Race/ethnicity			
Non-Hispanic White	45,462 (50.2)	2,005 (53.3)	0.09
Non-Hispanic Black	35,960 (39.7)	1,323 (35.2)	
Hispanic	5,002 (5.5)	236 (6.3)	
Other/missing	4,201 (4.6)	199 (5.3)	
Residence type			
Rural	23,729 (26.2)	930 (24.7)	0.03
Urban	66,896 (73.8)	2,833 (75.3)	
**Clinical characteristics**			
Body mass index			
Underweight	1,468 (1.6)	65 (1.7)	0.19
Normal	26,211 (28.9)	1,165 (31.0)	
Overweight	34,284 (37.8)	1,384 (36.8)	
Obese	27,866 (30.7)	1,019 (27.1)	
Missing	796 (0.9)	130 (3.5)	
Smoking status			
Never	11,214 (12.4)	481 (12.8)	0.12
Current	60,665 (66.9)	2,380 (63.2)	
Former	17,721 (19.6)	804 (21.4)	
Missing	1,025 (1.1)	98 (2.6)	
HIV co-infection	2,727 (3.0)	198 (5.3)	0.11
Charlson Comorbidity Index			
1	37,310 (41.2)	2,077 (55.2)	0.29
2	23,320 (25.7)	676 (18.0)	
3	8,821 (9.7)	277 (7.4)	
4	8,169 (9.0)	270 (7.2)	
≥5	13,005 (14.4)	463 (12.3)	
**Liver-related variables**			
HCV Genotype			

© 2023 Cartwright EJ et al. *JAMA Network Open.*

1	74,999 (82.8)	3,035 (80.7)	0.11
2	8,417 (9.3)	344 (9.1)	
3	4,974 (5.5)	234 (6.2)	
4/5/6	886 (1.0)	38 (1.0)	
Missing	1,349 (1.5)	112 (3.0)	
Hepatitis B co-infection	1,789 (2.0)	74 (2.0)	<0.01
Fibrosis 4 score			
<1.45	19,790 (21.8)	734 (19.5)	0.08
1.45-3.25	42,278 (46.7)	1,719 (45.7)	
>3.25	22,180 (24.5)	991 (26.3)	
Missing	6,377 (7.0)	319 (8.5)	
Hepatic decompensation	2,961 (3.3)	135 (3.6)	0.02
Liver cancer	1,933 (2.1)	125 (3.3)	0.07
**Treatment-related variables**			
Previous non-DAA therapy for HCV infection	13,572 (15.0)	585 (15.5)	0.02
Year of DAA therapy initiation			
2014	7,232 (8.0)	306 (8.1)	0.12
2015	25,304 (27.9)	968 (25.7)	
2016	33,651 (37.1)	1,289 (34.3)	
2017	18,659 (20.6)	880 (23.4)	
2018	5,779 (6.4)	320 (8.5)	
DAA regimen type			
Sofosbuvir/ledipasvir	53,024 (58.5)	2,200 (58.5)	0.08
Elbasvir/grazoprevir	8,923 (9.8)	317 (8.4)	
Sofosbuvir/velpatasvir ± voxileprevir	7,286 (8.0)	345 (9.2)	
Glecaprevir/pibrentasvir	3,609 (4.0)	184 (4.9)	
Paritaprevir/ritonavir/ombitasvir ± dasabuvir	6,996 (7.7)	282 (7.5)	
Sofosbuvir/daclatasvir	1,067 (1.2)	41 (1.1)	
Simeprevir/sofosbuvir	3,107 (3.4)	108 (2.9)	
Sofosbuvir/ribavirin	6,613 (7.3)	286 (7.6)	

Abbreviations: AUDIT-C, Alcohol Use Disorder Identification Test - Consumption; SMD, standardized mean difference; AUD, alcohol use disorder; IQR, interquartile range; HIV, human immunodeficiency virus; HCV, hepatitis C virus; DAA, direct-acting antiviral

Notes: All statistics reported as n(%) unless otherwise noted.

© 2023 Cartwright EJ et al. *JAMA Network Open.*

eTable 2. Covariate Distribution Between Those With and Without Outcome Data

	Not missing SVR n=76,568	Missing SVR n=14,057	SMD
**Exposure**			
Alcohol category			
Abstinent with no AUD	35,868 (46.8)	6,055 (43.1)	0.11
Abstinent with AUD	10,046 (13.1)	1,958 (13.9)	
Lower-risk consumption	14,879 (19.4)	2,632 (18.7)	
Moderate-risk consumption	3,489 (4.6)	629 (4.5)	
High-risk consumption or AUD	12,286 (16.0)	2,783 (19.8)	
**Demographics**			
Age in years, median (IQR)			
48-54	4,196 (5.5)	939 (6.7)	0.11
55-59	18,047 (23.6)	3,779 (26.9)	
60-64	29,474 (38.5)	5,367 (38.2)	
65-73	24,851 (32.5)	3,972 (28.3)	
Sex			
Women	2,295 (3.0)	419 (3.0)	<0.01
Men	74,273 (97.0)	13,638 (97.0)	
Race/ethnicity			
Non-Hispanic White	38,588 (50.4)	6,874 (48.9)	0.12
Non-Hispanic Black	30,577 (39.9)	5,383 (38.3)	
Hispanic	3,875 (5.1)	1,127 (8.0)	
Other/missing	3,528 (4.6)	673 (4.8)	
Residence type			
Rural	20,070 (26.2)	3,659 (26.0)	<0.01
Urban	56,498 (73.8)	10,398 (74.0)	
**Clinical characteristics**			
Body mass index			
Underweight	1,178 (1.5)	290 (2.1)	0.12
Normal	21,761 (28.4)	4,450 (31.7)	
Overweight	29,063 (38.0)	5,221 (37.1)	
Obese	23,969 (31.3)	3,897 (27.7)	
Missing	597 (0.8)	199 (1.4)	
Smoking status			
Never	9,691 (12.7)	1,523 (10.8)	0.15
Current	50,454 (65.9)	10,211 (72.6)	
Former	15,569 (20.3)	2,152 (15.3)	
Missing	854 (1.1)	171 (1.2)	
HIV co-infection	2,354 (3.1)	373 (2.7)	0.03
Charlson Comorbidity Index			
1	31,268 (40.8)	6,042 (43.0)	0.06
2	19,667 (25.7)	3,653 (26.0)	
3	7,468 (9.8)	1,353 (9.6)	
4	7,027 (9.2)	1,142 (8.1)	

© 2023 Cartwright EJ et al. *JAMA Network Open.*

≥5	11,138 (14.5)	1,867 (13.3)	
**Liver-related variables**			
HCV Genotype			
1	63,696 (83.2)	11,303 (80.4)	0.08
2	6,965 (9.1)	1,452 (10.3)	
3	4,106 (5.4)	868 (6.2)	
4/5/6	749 (1.0)	137 (1.0)	
Missing	1,052 (1.4)	297 (2.1)	
Hepatitis B co-infection	1,443 (1.9)	346 (2.5)	0.04
Fibrosis 4 score			
<1.45	16,566 (21.6)	3,224 (22.9)	0.06
1.45-3.25	36,068 (47.1)	6,210 (44.2)	
>3.25	18,656 (24.4)	3,524 (25.1)	
Missing	5,278 (6.9)	1,099 (7.8)	
Hepatic decompensation	2,426 (3.2)	535 (3.8)	0.03
Liver cancer	1,672 (2.2)	261 (1.9)	0.02
**Treatment-related variables**			
Previous non-DAA therapy for HCV infection	11,649 (15.2)	1,923 (13.7)	0.04
Year of DAA therapy initiation			
2014	6,305 (8.2)	927 (6.6)	0.19
2015	21,928 (28.6)	3,376 (24.0)	
2016	28,560 (37.3)	5,091 (36.2)	
2017	15,324 (20.0)	3,335 (23.7)	
2018	4,451 (5.8)	1,328 (9.4)	
DAA regimen type			
Sofosbuvir/ledipasvir	44,945 (58.7)	8,079 (57.5)	0.13
Elbasvir/grazoprevir	7,682 (10.0)	1,241 (8.8)	
Sofosbuvir/velpatasvir ± voxileprevir	5,941 (7.8)	1,345 (9.6)	
Glecaprevir/pibrentasvir	2,843 (3.7)	766 (5.4)	
Paritaprevir/ritonavir/ombitasvir ± dasabuvir	5,986 (7.8)	1,010 (7.2)	
Sofosbuvir/daclatasvir	895 (1.2)	172 (1.2)	
Simeprevir/sofosbuvir	2,748 (3.6)	359 (2.6)	
Sofosbuvir/ribavirin	5,528 (7.2)	1,085 (7.7)	

Abbreviations: SMD, standardized mean difference; AUD, alcohol use disorder; IQR, interquartile range; HIV, human immunodeficiency virus; HCV, hepatitis C virus; DAA, direct-acting antiviral
Notes: All statistics reported as n(%) unless otherwise noted.

© 2023 Cartwright EJ et al. *JAMA Network Open.*
