# Supplementary material for: Alcohol Use and Sustained Virologic Response to Hepatitis C Virus Direct-Acting Antiviral Therapy
Source: JAMA Netw Open. 2023 Sep 26;6(9):e2335715. doi: 10.1001/jamanetworkopen.2023.35715 (PMC10523171; doi:10.1001/jamanetworkopen.2023.35715)
Supplement: Supplement 2. — Data Sharing Statement [file jamanetwopen-e2335715-s002.pdf]

Data Sharing Statement

Cartwright. Alcohol Use and Sustained Virologic Response to Hepatitis C Virus Direct-Acting Antiviral Therapy. *JAMA Netw Open*. Published September 26, 2023. doi:10.1001/jamanetworkopen.2023.35715

Data
Data available: No

Additional Information
Explanation for why data not available: Due to US Department of Veterans Affairs (VA) regulations and our ethics agreements, the analytic data sets used for this study are not permitted to leave the VA firewall without a data use agreement. This limitation is consistent with other studies based on VA data. However, VA data are made freely available to researchers with an approved VA study protocol. For more information, please visit https://www.virec.research.va.gov or contact the VA Information Resource Center at VIReC@va.gov.
